# Family planning to promote physical activity: a randomized controlled trial protocol

**DOI:** 10.1186/s12889-015-2309-x

**Published:** 2015-10-05

**Authors:** Alison Quinlan, Ryan E. Rhodes, Chris M. Blanchard, Patti-Jean Naylor, Darren E.R. Warburton

**Affiliations:** Behavioural Medicine Laboratory, University of Victoria, 3800 Finnerty Rd., Victoria, BC V8P-5C2 Canada; Dalhousie University, Halifax, NS Canada; University of British, Columbia, BC Canada

**Keywords:** Action Control, Children, Parents, Exercise, Intention-behavior gap

## Abstract

**Background:**

Physical activity is associated with the reduction of several chronic conditions in adults. Additionally, physical activity is extremely important for children for their development and cognitive functioning and also to create a physically active lifestyle that continues into adulthood. Despite the known benefits of physical activity, only one in five adults are achieving the public health recommendations of 150 minutes of moderate-to-vigorous physical activity per week and only 13 % of boys and 6 % of girls between the ages of 5 and 17 years are meeting the guidelines of 60 minutes per day. This study aims to evaluate whether a planning condition improves adherence to regular physical activity compared to an education-only control condition among families. Families are eligible if there is at least one child between the ages of 6 and 12 years who is not meeting the Canadian Physical Activity Guidelines.

**Methods/design:**

A six-month longitudinal randomized controlled trial will be used to compare the two conditions. Materials will be delivered at baseline with ‘booster’ sessions at six weeks and three months. Participants will be assessed at baseline and at six months with a fitness test, as well as questionnaires and accelerometery at baseline, six weeks, three months and six months. A total of 137 families have been recruited thus far from Greater Victoria. This study is ongoing and recruitment will continue until December 2015 with the target goal of reaching 160 families.

**Discussion:**

This protocol describes the implementation of a randomized controlled trial that utilizes planning strategies to try and increase physical activity among families. Research findings could be useful in public health in providing effective strategies to families to help decrease sedentary lifestyles. Additionally, findings may help to inform future interventions aimed at increasing physical activity among families.

**Trial registration:**

This trial was registered on June 5, 2012 with the Clinical Trials Registry maintained by the National Library of Medicine at the National Institutes of Health. The registration ID is NCT01882192.

## Background

Recent reports suggest that sedentary lifestyles are rapidly increasing which pose as a serious risk factor for many adverse health outcomes. Sure enough, the burden of chronic disease is increasing as are obesity rates. According to the most recent data from the World Health Organization and Statistics Canada, the two leading causes of death are from heart disease and cancer with approximately 29.9 % of Canadian adults dying from cancer and 19.7 % from heart disease [1]. Additionally, obesity rates are on the rise. Almost 60 % of Canadians are either overweight or obese, and this has become an ever-increasing trend [2, 3]. This is also the case with children. Approximately one third (31.5 %) of Canadian youth aged 5 to 17 years old are overweight or obese [2, 4]. Obesity is rapidly becoming one of the more serious public health challenges of this century. The need for changes to modifiable risk factors associated with obesity and chronic diseases is paramount.

Physical activity (PA) is associated with the reduction of several chronic diseases in adults, including breast cancer, colorectal cancer, cardiovascular disease (CVD), stroke, high blood pressure, type 2 diabetes, osteoporosis, and hypertension [5]. Furthermore, there is a dose–response relationship between physical activity and these disease states, which means that the more activity performed, the greater the health benefits, such as reducing the risk of premature all-cause mortality [5]. In fact, physically active individuals have an approximate risk reduction of 31 % - 45 % for premature all-cause mortality compared to physically inactive individuals [5]. Physical activity can provide numerous benefits for children as well. In children 5 to 17 years old, physical activity and high physical fitness help guard against high blood pressure, high blood cholesterol, metabolic syndrome, low bone density, depression, injuries, and obesity [6]. Despite these well-known benefits, results from the latest Canadian Health Measures Survey (CHMS) brought to light some alarming statistics. Adults aged 18 to 79 accumulated on average of 12 minutes per day of moderate-to-vigorous physical activity for a total of 84 minutes per week [7]. Current recommended guidelines suggest adults achieve 150 minutes of moderate-to-vigorous physical activity (MVPA) per week working out to about 30 minutes a day five times a week [8]. Only about 1 in every 5 adults are achieving these public health recommendations. Even more alarming were the results for Canadian children showing that for kids between the ages of 5 and 17 only 13 % of boys and 6 % of girls were achieving the guidelines of 60 minutes a day of MVPA [9].

It is widely accepted that children and youth are influenced by the settings they spend their time in and by the adults in those settings. The home is a key environment and parents are the adults that typically govern health promotion choices as children are not cognitively prepared to make decisions that influence long-term health outcomes [10]. There has been significant research on parental correlates of PA among children. For example, in a recent review of reviews by Rhodes and Quinlan [11], they report on over 100 studies that have focused on family influence and child physical activity. Among several types of family correlates (e.g., parental role modeling, parental support, parental attitudes about PA, general parenting styles and overall family cohesion), parental support has the most clear and consistent evidence as a correlate of child PA.

Obviously, regular life-long physical activity is the desired strategy for reducing the risk of chronic diseases and obesity; thus promotion efforts should be targeting families due to the influence parents have on their children’s physical activity levels as well as the decline in PA for parents [11].

### Theoretical framework

Unfortunately, while parental support is a key correlate of child physical activity, few interventions at the family-level have yielded behavior change outcomes [12]. In order for interventions to be effective in changing behavior, it has been suggested that a sound understanding of physical activity determinants, preferably via a theoretical framework is needed [13].

The majority of the interventions that have been conducted involved educational materials on the benefits of child and family physical activity, which may not effectively mediate parental support [12]. For example, Rhodes and colleagues [14] used an adapted theory of planned behavior model [15] to understand parental support of child physical activity and showed that most parents already viewed physical activity as very important for their children. Thus, attitudes about the benefits of child physical activity had no correlation with child physical activity due to this ceiling effect. Conversely, other research by Rhodes et al. [16] showed that parental self-regulation skills – particularly planning, monitoring, and seeking out opportunities for child activity - were critical to child physical activity. Our research complements several researchers/theoreticians who have begun to expand traditional social cognitive theories, focusing on increasing attitudes and control into motivational and volitional planning phases for successful physical activity adherence [17–20].

The Multi-Process Action Control Framework (M-PAC) [19, 21] is more in line with these approaches. In this framework, parents intending to support family physical activity for their children are thought to be influenced by affective attitude (enjoyment of the behavior) and perceived control (ability and opportunity to perform the behavior if desired), and are largely dependent on the higher use and quality of regulation behaviors (e.g., planning, seeking opportunities, self-monitoring). Therefore, the purpose of this paper is to describe the protocol for a family physical activity intervention guided by M-PAC and focused on building high quality planning strategies to create opportunities to enact fun physical activities.

### Pilot research

This research builds on a pilot study conducted from January 2007 until December 2008 in the Capitol Region District of British Columbia that intervened with 85 families [22]. The measures, intervention protocol, recruitment, training of personnel, and related practicalities were all tested during the pilot. The pilot work was successful in demonstrating both sustained recruitment and expected outcomes. As hypothesized, the planning intervention resulted in higher family physical activity compared to the standard condition (effect size η = .08 to .11). Furthermore, the results showed no changes to intention or perceived behavioral control, supporting the concept of a self-regulatory planning phase in physical activity adherence. While these findings were promising, the study was pilot in nature and thus the current trial represents an extension of the work; increasing the methodological veracity and the sophistication of the intervention. First, this study improves upon the pilot study by using direct assessment of physical activity (i.e., accelerometry) rather than self-report, which is the recommended form of assessment for these population groups [23, 24]. Second, this study will evaluate the efficacy of the intervention across six months rather than the one-month time frame used in the pilot. Third, the current study provides a more comprehensive planning skills intervention package based on contemporary theory [20, 21]. Fourth, the study will include health-related assessments of quality of life and physical fitness which were absent in the pilot. These assessments are important markers of health benefits in addition to physical activity and help ascertain whether any changes in behavior actually link to the sought after health improvements that form the central rationale for physical activity promotion.

### Present research

The primary research question is whether a planning condition improves adherence to regular physical activity compared to a control condition at six months. We have four secondary research questions that will also be examined:

1. Does the planning condition improve motivational, health-related quality of life, and health-related fitness outcomes compared to the control condition at six months?
2. Can group differences among these motivational, behavioural, and health-related fitness outcomes be explained through a mediation model?
3. Can motivational variables predict adherence? Do these differ by condition? Our hypothesis is that
4. Is there an intergenerational, seasonal, or gender difference across primary outcomes by assigned condition?

### Hypotheses

We hypothesize that adherence will be higher for the planning condition in comparison to the more standard physical activity education condition. We also propose that the effect may wane over time from the initial measurement period but all outcomes will remain significantly higher at six months. We hypothesize that the planning condition will not affect intentions but will positively affect health related fitness and QOL. We also hypothesize that the covariance of the assigned conditions (planning, education) on use/adherence will be explained by planning and the use of behavioral regulation strategies (i.e., manipulation check). In turn, the covariance between planning and behavioral regulation strategies and health-related outcomes will be explained by physical activity adherence among conditions. In addition, affective attitude and perceived behavioral control will predict intention, intention will predict planning and planning will predict adherence across conditions. Finally, we hypothesize that children will show greater adherence to the planning condition than their parents. No differences in gender or season are hypothesized but these are exploratory research questions because there is limited research at present to make any definitive statement.

## Methods/design

### Participants and recruitment procedure

Families residing in Victoria, British Columbia with at least one parent and at least one child between the ages of six and 12 years who are not currently meeting physical activity guidelines over the last three months (i.e. parents should accrue at least 150 min of moderate-to-vigorous intensity activity per week (e.g., 5 days x 30 min) and children 60 min of activity daily [8] will be included (screened by self-report through initial recruitment contact interview). We will also conduct a secondary inclusion screen based on baseline accelerometry results which are the golden standard for physical activity assessment [23, 24]. Those families with children who are above the Canadian recommended guidelines that were initially screened into the trial based on self-report will be excluded from the trial. This age group was selected based on earlier pilot work [22] and the fact that children under six years of age engage in physical activity that is extremely sporadic, and thus very different than adults [25]. Our decision to limit the age of children to 12 was based on more pragmatic grounds; in this case, 12 year old children represent the upper bound of the “tweens”, where parents are still very influential in physical activity decisions and planning interventions at the level of the parent would be most effective [10, 12].

### Recruitment

Based on our pilot study recruitment [22], participants will be recruited via advertisements placed in local newspapers, through schools, recreation centres, health care centres, children’s recreation classes, shopping malls, and outdoor markets. In order to ensure diversity in the study population, recruitment is performed by stratifying the city into regions. Within each region, a complete list of schools, recreation centres, health care centres, children’s recreation classes, outdoor markets and shopping malls are obtained and the same number of each type of facility in each region is randomly selected and contacted for recruitment. We currently recruit approximately 5–10 families per week with this strategy. Additionally we incorporated a participant incentive program for snowball recruitment. Any family who refers another family and they enroll in the study is eligible for a $25 grocery store gift card.

### Design

This study is a two-arm parallel design single blinded randomized controlled trial. Participants will be randomised to one of two groups 1) physical activity planning condition; or 2) standard physical activity education condition for six months duration. The RCT has been registered with with the Clinical Trials Registry maintained by the National Library of Medicine at the National Institutes of Health.

### Randomization

Prior to the baseline fitness test, written informed consent will be obtained from the parents, and verbal informed consent will be obtained from the children. A research assistant will explain to the child what is required to participate and the child has to verbally say that they understand and are willing to participate. If a child says no, or remains silent, they will not be eligible to participate in the study. After the baseline fitness test, families (both parents and all children between ages 6–12 years) will be asked to wear accelerometers for one week and will be instructed on how to enter information about each day’s activity into a log. Research assistants will provide a short training session on how to wear and use the accelerometers. After wearing the accelerometer for 7 days, families will be randomized at a 1:1 ratio to either the intervention or control group, using a central computerized system. Participants will be aware of their group allocation, but the fitness testers will be blinded to the treatment allocation. At the end of the week of wear, a research assistant will come to the families’ homes to pick up the accelerometers and go through the study materials and thus the research assistant will not be blind to the families’ condition.

### Intervention

We will be comparing a standard information condition to an information plus planning condition. The standard (comparison group) package will consist of Canada’s physical activity guidelines [8] recommending 60 minutes of activity a day in bouts as short as five to ten minutes for children and a breakdown of ways for the family to achieve this physical activity (structured, unstructured, endurance, strength, activities, less than 60 minutes of sustained sedentary activity, reduce screen viewing by 30 min per day) commensurate with this guide. This will include the insert by CSEP [8]. The intervention also includes an adapted information booklet that we designed that emphasizes the benefits of physical activity and provides some strategies for working through common barriers to being active. The intervention condition will receive the same guidelines as the comparison condition but will also be provided with family physical activity planning material. This material will include skill training content (workbook how to plan for family physical activity) and practical material to create a plan (i.e., a colorful dry erase wall calendar for family activities with fridge magnets). The skill training material for planning is based on several streams of prior work in the adult physical activity literature. Families are instructed to plan for “when,” “where,” “how,” and “what” physical activity will be performed commensurate with the creation of implementation intentions/action planning [e.g., [26, 27]]. The workbook, however, also focuses on problem solving barriers to physical activity which is more akin to coping planning and traditional goal setting [28–31]. The design of all material was tested during implementation of the pilot and features graphic design and color images that represent family physical activity.

## Outcome measures

### Primary outcome measure

#### Change from baseline in children’s physical activity to 6 months

Children’s physical activity will be assessed at four separate time points: baseline, six-weeks, three-months and six-months. Children’s physical activity will be quantified by accelerometry. Children will wear a GT3X accelerometer on their right hip for a minimum of 10 hours per day for 7 days at each time point [32, 33].

### Secondary outcomes include

#### Parent’s physical activity

Parent’s physical activity will be quantified by accelerometry. Parents will wear an accelerometer for 7 days at baseline, six-weeks, three and six-months, for a minimum of 10 hours per day [33].

#### Motivation

Motivations for family based physical activity and personal physical activity will be measured using the constructs of the theory of planned behavior [15] including intention affective attitude, instrumental attitude, perceived control, adapted within the M-PAC framework [21]. Our measures of the theory of planned behaviour are validated from our previous research and have shown excellent predictive validity and internal consistency in adult [14, 16, 34] and child/adolescent [35] populations. These have been validated for both personal and family-based (i.e., activities as a family collective) physical activity [34]. Items will measure all components of the model (affective attitude, instrumental attitude, injunctive norm, descriptive norm, perceived control) including behavioural, normative, and control beliefs developed from prior pilot work in families. There will also be a measurement of habit [36] and identity [37] commensurate with the M-PAC model. The questionnaire will be completed by both parents and the target child. Change in motivation variables will be examined at baseline, six-weeks, three-months and six-months.

#### Self-reported family based physical activity and personal physical activity

All children will complete a modified version of the Physical Activity Questionnaire for Children (PAQ-C) [38] to assess habitual moderate to vigorous physical activity. The Godin Leisure-Time Exercise Questionnaire (LSI) will be used to measure self-reported physical activity in parents [39]. The LSI contains three questions, which assess the frequency of mild, moderate, and strenuous activity performed for at least 15 minutes during free time in a typical week. Self-reported physical activity will be examined at baseline, six-weeks, three-months, and six-months.

#### Health-related quality of life / psychosocial distress

Health-related quality of life / psychosocial distress will be assessed with parents using the Satisfaction with Life Scale [40]. The instrument is well-known and widely used.

Children’s Quality of Life will be assessed using the 5-item Satisfaction with Life Scale Adapted for Children (SWLS-C) [41]. The SWLS-C was adapted from the Satisfaction with Life Scale. The SWLS-C demonstrates a unidimensional factor structure and sound internal consistency [41].

#### Body composition

Skinfolds (triceps, biceps, subscapular, supra iliac, medial calf) will be measured using standard anthropometric procedures. Change in body mass index (BMI), waist circumference, and sum of 5 sites will be examined from baseline to 6 months (post-intervention).

#### Cardiovascular fitness

A steady-state walking treadmill test will be used to assess cardiovascular fitness in both parents and children. Heart rate, and blood pressure (sphygmomanometer and a stethoscope) will be monitored at rest and during exercise.

#### Musculoskeletal fitness

Grip strength, push ups, sit & reach flexibility, partial curl-ups, vertical jump and back extension will be measured to determine the musculoskeletal fitness of both the children and parents using the Canadian CSEP standardized protocols. Change in musculoskeletal fitness from baseline to 6 months (i.e., post-intervention) will be examined.

#### Demographics

A brief section in the baseline questionnaire will be used to assess characteristics including age, gender, marital status, ethnicity, level of education, health background, employment information, sleep habits, smoking habits, alcohol habits and general nutrition habits. We will use measures developed from a recently completed longitudinal study of parents [42].

The sole use of quantitative methods to understand the low physical activity in specific populations has been criticized. It has been suggested that other approaches be utilized to gain a more in-depth understanding of the issue [43–45]. Although quantitative measurement of outcomes will provide insight into the potency of our intervention, a process evaluation (whereby participants are interviewed) is also essential to examine the *content fidelity* (“what is done”) and *process fidelity* (“how it is done”) of program implementation [46]. Furthermore, as Plumer and colleagues [47] suggest, process evaluations “can help explain the program’s outcomes and identify ways to improve and/or replicate it. This will be accomplished through a member of the research team conducting a ten-minute interview with each family at the end of the study. Questions asked will start off broad asking the family to discuss the importance of family based physical activity, if they noticed any changes over the length of the study, and they move more specifically to the materials asking them what they liked/disliked about the study materials, what they found helpful/not helpful from the study materials and an opportunity to provide any additional thoughts or suggestions on what they felt could have been helpful to increase physical activity.

### Analysis strategy

Data will be evaluated for patterns of missingness for each psychosocial variable and behavior at all time points using the dummy coding procedures of Allison [48]. Depending on the outcome of these tests (e.g., missing at random, missing completely at random, etc.) we will initiate the appropriate missing data handling strategy. ITT analyses will also be performed in addition to sensitivity analysis procedures. Our first research question will be analyzed using a 2 (condition) x 4 (time) repeated measures factorial ANOVA on the primary outcome of child adherence. A child from each household in the eligibility range will serve for this analysis (chosen a priori through randomization procedures). Post hoc examinations using Tukey follow-up procedures will be utilized if necessary. Our secondary research questions (parent; parent/child; gender; season; fitness variables, etc.), will also be analyzed using a variant of this design with the addition of factors. Cluster analysis/HLM will be used for parent/child collinearity [49]. Our pilot study (r = .21) and prior research [10] suggests limited collinearity but it is appropriate to explore findings with these approaches given these are naturally clustered environments (i.e., family home) [49]. The qualitative analyses will incorporate the following processes [50, 51]: 1) Invite participants to review the transcripts of interviews, and summarize their perception of the data, for accuracy, and check for the trustworthiness of the data; 2) Conduct a thematic analysis using a reciprocal coding approach where researchers engage in open dialogue about themes and data interpretation. In doing so, each transcript is first reviewed independently, then through dialogue composite themes and related critical issues are developed; and 3) Manage the data using the NVivo software program. NVivo enables theory building, testing and elaboration. With NVivo, ‘free nodes’ can be created during the coding process, capturing participants’ perspectives and the investigators’ critical issues.

### Statistical power and sample size

Based on our previous research, **160** families (80 per group) will be recruited to detect a small-medium effect size (f^2^ = .10; [52]) in adherence to physical activity (primary outcome) with a type one error of .05, an average correlation of .75 across time for our DV of interest, and a power of .80. Our sample size also considers the main 2 (group) x 2 (parent/child) x 4 (time) repeated measures design using G-Power [53] and a potential 25 % attrition rate similar to the pilot study. Finally, the evaluation of physiological outcomes of participants across time will follow a 2 (condition) x 2 (time) interaction. Physiological parameters have larger effect sizes than the social/psychological variables in the study due to smaller standard error scores. The proposed sample size is, therefore, more than adequate to ensure sufficient statistical power for the physiological measurements.

## Results

**Fig. 1:**
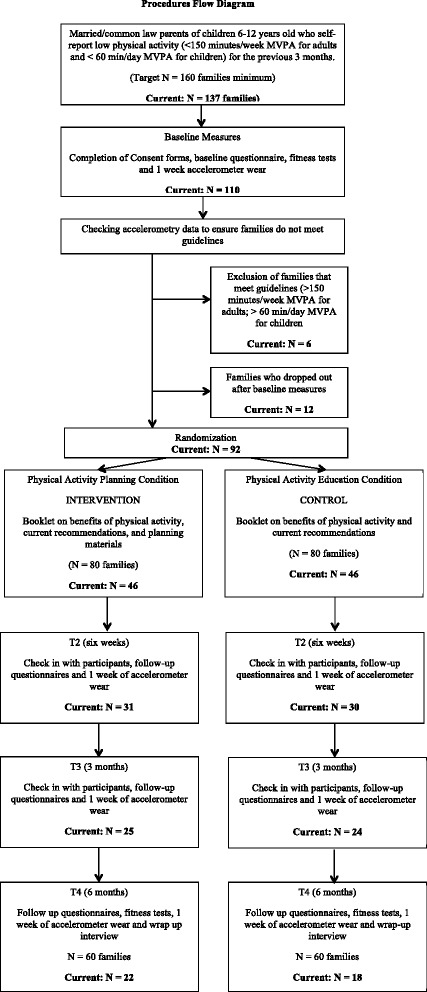
Study Procedures and Participant Flow Diagram

To date, we have obtained ethical approval, registered the trial and have 137 families recruited from the Greater Victoria region. Ethical approval was received from the University of Victoria Human Research Ethics Board. We aim to complete recruitment by December 2015. From the 137 families recruited, 110 have completed all of the baseline measures, 62 have completed the six-week measures, 50 have completed the three month measures, and 40 families have completed the study. The study is ongoing and data analysis will continue into 2016. Please see Fig. 1 for the study procedures and participant flow chart.

## Discussion

This protocol describes the implementation of a randomized controlled trial that utilizes planning strategies to try and increase physical activity among families based on the assumptions of the M-PAC framework as a conceptual model. Research findings will be important to public health as they may help to determine if providing low-cost, scalable and evidence-based planning strategies for family physical activity can aid in producing higher adherence to physical activity.
